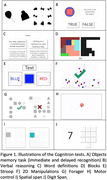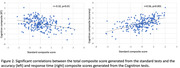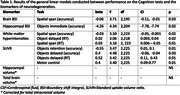# Remote cognitive testing in Insight46: relationship to standard cognitive assessments and biomarkers of neurodegeneration

**DOI:** 10.1002/alz.088713

**Published:** 2025-01-03

**Authors:** Martina Del Giovane, Marguerite Leoni, Valentina Giunchiglia, Ziyuan Cai, Rebecca E Street, Kirsty Lu, Andrew Wong, Maria Popham, Insight 46 study Team, Heidi Murray‐Smith, Marcus Richards, Sebastian J Crutch, Adam Hampshire, Jonathan M Schott

**Affiliations:** ^1^ Imperial College London and the University of Surrey, UK Dementia Research Institute Care Research and Technology Centre, London, London United Kingdom; ^2^ Imperial College London, London, London United Kingdom; ^3^ Imperial College London, Department of Brain Sciences, London United Kingdom; ^4^ Dementia Research Centre, UCL Queen Square Institute of Neurology, University College London, London United Kingdom; ^5^ MRC Unit for Lifelong Health and Ageing at UCL, London United Kingdom; ^6^ Dementia Research Centre, UCL Queen Square Institute of Neurology, London United Kingdom; ^7^ MRC Unit for Lifelong Health & Ageing at UCL, London United Kingdom

## Abstract

**Background:**

Dementia‐related biomarkers can detect pathology years before clinical diagnostic criteria are met. Understanding the relationship between biomarkers and early cognitive changes is crucial as disease‐modifying therapies may have maximum benefits when delivered early. We aimed to demonstrate the utility of remote computerised cognitive tests in a large cohort of cognitively normal older individuals, comparing these to standard in‐person assessments and investigating their associations with biomarkers.

**Methods:**

Using the Cognitron platform, we remotely deployed 11 computerised tests (Figure 1) in 255 members of the Insight46 study. Participants had previously completed biomarker and in‐person standard cognitive assessments at age 71‐73 (1). We generated brain, hippocampal, and white matter hyperintensity volumes, rates of brain and hippocampal volume change between age 71‐73, amyloid load (SUVR) and positivity as previously described (1). General linear models (GLMs) assessed the relationship between the Cognitron tests and biomarkers. Principal component analysis was used for dimensionality reduction, and bivariate correlations compared the standard tests total composite score with the Cognitron accuracy and response time (RT) composite scores. Canonical Correlation Analysis (CCA) further quantified these associations.

**Results:**

Of 255 participants (132 males, 234 right‐handed, age at assessment 77), 66 were amyloid positive. Amyloid positivity was significantly predicted by RT for the Objects memory test (delayed recognition) (OR = 1.58 (95% CI 0.09,0.85)). We found an association between the Cognitron Objects memory tests and biomarkers (Table 1). Immediate recognition was significantly associated with hippocampal volume change (F_(2,204_) = 4.34; CI: ‐7.78, ‐7.74), while the amount of amyloid positivity was significantly predicted by delayed recognition (F_(2,225) =_ 4.48; CI: ‐0.06, ‐0.04) and retention (F_(2,204) =_ 3.47; CI: 0.01, 0.1). Both Cognitron accuracy and RT composites significantly correlated with the standard composite (r = 0.56, p<0.001; r = ‐0.32, p<0.001) (Figure 2). CCA indicated shared variance with significant canonical variables between the standard tests and the Cognitron accuracy (M1:r = 0.73, p<0.001; M2:r = 0.54, p<0.001; M3:r = 0.49, p = 0.01) and RT (M1:r = 0.54, p<0.001) scores.

**Conclusions:**

Remote computerised cognitive testing correlates with standard supervised assessments and holds potential for studying early cognitive changes associated with neurodegenerative biomarkers at scale and longitudinally.

**Reference**

1. Lane et al. (2017) ‐ *BMC Neurol*, 17(75). https://doi.org/10.1186/s12883‐017‐0846‐x